# Comparative transcriptome profiling and co-expression network analysis uncover the key genes associated with pear petal defense responses against *Monilinia laxa* infection

**DOI:** 10.3389/fpls.2024.1377937

**Published:** 2024-03-07

**Authors:** Meriem Miyassa Aci, Polina C. Tsalgatidou, Anastasia Boutsika, Andreas Dalianis, Maria Michaliou, Costas Delis, Dimitrios I. Tsitsigiannis, Epaminondas Paplomatas, Antonino Malacrinò, Leonardo Schena, Antonios Zambounis

**Affiliations:** ^1^ Department of Agriculture, Università degli Studi Mediterranea di Reggio Calabria, Reggio Calabria, Italy; ^2^ Department of Agriculture, University of the Peloponnese, Kalamata, Greece; ^3^ Institute of Plant Breeding and Genetic Resources, Hellenic Agricultural Organization Dimitra, Thessaloniki, Greece; ^4^ Laboratory of Vegetable Crops, Institute of Olive Tree, Subtropical Crops and Viticulture, Hellenic Agricultural Organization Dimitra, Heraklion, Greece; ^5^ Laboratory of Plant Pathology, Department of Crop Science, Agricultural University of Athens, Athens, Greece

**Keywords:** pear flower, RNA-seq, WGCNA, regulatory networks, immune response, pathogens, transcription factors

## Abstract

Pear brown rot and blossom blight caused by *Monilinia laxa* seriously affect pear production worldwide. Here, we compared the transcriptomic profiles of petals after inoculation with *M. laxa* using two pear cultivars with different levels of sensitivity to disease (Sissy, a relatively tolerant cultivar, and Kristalli, a highly susceptible cultivar). Physiological indexes were also monitored in the petals of both cultivars at 2 h and 48 h after infection (2 HAI and 48 HAI). RNA-seq data and weighted gene co-expression network analysis (WGCNA) allowed the identification of key genes and pathways involved in immune- and defense-related responses that were specific for each cultivar in a time-dependent manner. In particular, in the Kristalli cultivar, a significant transcriptome reprogramming occurred early at 2 HAI and was accompanied either by suppression of key differentially expressed genes (DEGs) involved in the modulation of any defense responses or by activation of DEGs acting as sensitivity factors promoting susceptibility. In contrast to the considerably high number of DEGs induced early in the Kristalli cultivar, upregulation of specific DEGs involved in pathogen perception and signal transduction, biosynthesis of secondary and primary metabolism, and other defense-related responses was delayed in the Sissy cultivar, occurring at 48 HAI. The WGCNA highlighted one module that was significantly and highly correlated to the relatively tolerant cultivar. Six hub genes were identified within this module, including three WRKY transcription factor-encoding genes: *WRKY 65* (pycom05g27470), *WRKY* 71 (pycom10g22220), and *WRKY28* (pycom17g13130), which may play a crucial role in enhancing the tolerance of pear petals to *M. laxa*. Our results will provide insights into the interplay of the molecular mechanisms underlying immune responses of petals at the pear–*M. laxa* pathosystem.

## Introduction

Pear (*Pyrus communis* L.) cultivation, a significant component of the global fruit industry (Kang et al., 2021), faces significant challenges due to susceptibility to various pathogens, including *Monilinia laxa* (Aderhold & Ruhland) Honey (Petróczy et al., 2012; Martini and Mari, 2014). Commonly known as a causal agent of the brown rot disease, this necrotrophic fungus spreads rapidly in pear orchards (Ogawa and English, 1991; Petróczy et al., 2012; Sardella et al., 2016). The pathogen infects flowers during their vulnerable blooming stage and causes extensive fruit rots in late season resulting in substantial economic losses (Martini and Mari, 2014).

To defend against fungal infections, plants have developed a complex repertoire of innate immune responses (Saijo et al., 2018). Particularly upon challenge with necrotrophs, a fine-tuned layer of cell surface immune receptors initiates basal defenses known as pattern-triggered immunity (PTI) responses (Couto and Zipfel, 2016). In addition, even when PTI is overwhelmed, an alternative cell wall integrity mechanism may be activated to induce defense responses upon fungal challenge (Engelsdorf et al., 2018). Previous studies have highlighted the molecular mechanisms implemented by both *M. laxa* and stone fruits during the early infection stages (Balsells-Llauradó et al., 2020; Villarino et al., 2022). Thus, activation of the carbohydrate-active enzyme (CAZyme)-encoding genes is employed by *M. laxa* to achieve penetration (Balsells-Llauradó et al., 2020), whereas non-ribosomal peptide synthase (*NRPS*)-encoding genes are potentially involved in the production of fungal toxins during the colonization of fruit tissues (Villarino et al., 2022). On the other hand, the host molecular mechanisms against *M. laxa* were previously reported using comparative transcriptome analysis between resistant and sensitive peach genotypes, highlighting the involvement of hormone signal transduction, phenylpropanoid, flavonoid, and glutathione metabolismrelated genes in defense responses (Guidarelli et al., 2014; Balsells-Llauradó et al., 2020). Furthermore, many transcription factor (TF)-encoding genes, such as members of the *WRKY*, *MYB*, *ERF*, and *NAC* families, have been suggested to be involved in mediating the immune responses of fruits against *M. laxa* (Guidarelli et al., 2014; Balsells-Llauradó et al., 2020).

Despite the considerable losses due to blossom blight caused by *M. laxa*, the molecular interplay between this necrotrophic fungus and pear petals has not yet been deciphered. Previously, it was recorded that 19, 35, and 7 WRKY-encoding genes were involved in the regulation of defense responses in rose petals, grapevine, and strawberry flowers to *Botrytis cinerea*, respectively (Haile et al., 2017; Liu et al., 2018; Xiao et al., 2022). TFs are key regulators of the host transcriptional reprogramming during pathogen attack (Pandey and Somssich, 2009; Phukan et al., 2016), and among them, WRKYs are the most involved TF family in plant defense regulation (Zhang et al., 2016; Yuan et al., 2018; Zambounis et al., 2020; Xiao et al., 2022). Furthermore, the positive impact of WRKY TFs in rice and *Arabidopsis* responses to pathogen attacks was further highlighted as WRKYs can functionally cooperate in co-regulatory networks such as in the “COR-A” network (Berri et al., 2009).

In plant–pathogen interactions, the regulation of defense processes is usually mediated by the induction of a massive reprogramming, which is redirected to involve highly connected and complex molecular networks (Delplace et al., 2022). In order to reveal a detailed framework of the host responses to pathogen attack, the identification of the main regulators (hub genes) that orchestrate the transcriptional reprogramming beyond plant defense responses can be achieved through powerful system biology approaches such as weighted gene co-expression network analyses (WGCNA). Hub genes allow the identification of key counterparts with high connectivity degree in an interactive gene network, which usually play important roles in the regulation of several other genes and biological processes (Yu et al., 2017).

In this study, we compared the transcriptome dynamics of pear petals at two early stages of *M. laxa* infection between two cultivars characterized by different levels of sensitivity to this pathogen. We particularly explored the transcriptional network governing responses to *M. laxa* infection to uncover the molecular mechanisms underlying various aspects of immune responses. Specific differentially expressed genes (DEGs) involved in cell wall modification processes, pathogen recognition, and hormone signal transduction contributed to the differential and time-dependent defense-related responses among the two cultivars. The role of regulatory TFs associated with these responses in the relatively tolerant cultivar was also revealed by constructing co-expression regulatory networks to identify key genes regulating this interplay. These genes may be potential targets in future breeding programs toward a sustainable disease management strategy against *M. laxa* in pear.

## Materials and methods

### Plant material

Two pear (*Pyrus communis* L.) cultivars (cv. Sissy with relative tolerance and cv. Kristalli with high sensitivity to fungal diseases) were grown in a greenhouse under the following conditions: 20°C–25°C, 50%–70% relative humidity, and 16:8 h light/dark photoperiod. The two cultivars (cv. Sissy and cv. Kristalli) were grafted on BA29 and OHxF333 rootstocks, respectively. Intact pear flowers without any visible signs of brown rot were collected in blossom (fully open buds) and placed with their stems in polystyrene boxes filled with deionized water under controlled conditions (23°C–25°C with 30%–40% relative humidity and 12:12 h photoperiod). Petals were gently detached from the flowers, disinfected by dipping in a 1% sodium hypochlorite solution for 5 min, rinsed three times with sterile deionized water, air-dried in a laminar flow hood under sterilized conditions, and placed on 0.4% water agar with 10 petals per Petri dish.

### Pathogen inoculation

A virulent *M. laxa* strain (isolate no. 1387) was kindly provided by the fungal collection of Benaki Phytopathological Institute (Athens, Greece). Conidia from 10-day-old potato dextrose agar (PDA) cultures were suspended in potato dextrose broth (PDB), adjusted to the concentration of 10^6^ conidia/ml and used to inoculate petals of both cultivars by dropping 10-μl drops onto the central adaxial surface of each petal (ML). Petals mock inoculated with PDB were used as controls (CT). Petal disks were retrieved by cutting sections of 5 mm diameter around the inoculation sites at 2 HAI or 48 HAI. All petal discs were instantly frozen in liquid nitrogen and stored at −80°C until use. Petal disks were retrieved by cutting sections of 5 mm diameter around the inoculation sites at 2 HAI or 48 HAI. All petal discs were instantly frozen in liquid nitrogen and stored at −80°C until use. A total of eight petal treatments were performed for the RNA-seq analysis and the other assays, named as S2CT, S2ML, S48CT, and S48ML for the Sissy (S) cultivar and K2CT, K2ML, K48CT, and K48ML for the Kristalli (K) cultivar across the two time points. All treatments included three biological replicates, each consisting of 20 pooled petals (480 petals in total).

### Disease severity assays

The size of brown rot necrotic lesions that developed on the petals upon ML treatments of both cultivars at 48 HAI was categorized using the following disease index scale: 0, no infection; 1, lesion covering <10%; 2, lesion covering 11%–25%; 3, lesion covering 26%–50%; 4, lesion covering 51%–75%; and 5, lesion covering >75% of the petal. Lesion diameters were also recorded in the petals of both cultivars at 48 HAI. Furthermore, to evaluate the relative sensitivity of each cultivar against *M. laxa*, stamens of flowers with petals removed were evenly spray-inoculated with a conidial suspension of 10^6^ conidia/ml. The disease symptoms of the inoculated flowers were evaluated at 120 h after infection by transferring them into sterile humidity chambers at 22°C. Three independent experiments were performed each consisting of 10 flowers of each cultivar. Statistical analysis was performed using the IBM SPSS Statistics, version 25.0 (IBM Corp., Armonk, NY, USA) with ANOVA followed by the non-parametric Mann–Whitney *U*-test (*p* = 0.05) for disease index calculation, whereas a *t*-test for independent samples (*p* = 0.05) was used for the lesion diameter recording.

### Lipid peroxidation and hydrogen peroxide assays on pear petals

An amount of 150 mg of fresh petal material was homogenized in liquid nitrogen, diluted with 0.1% trichloroacetic acid at 4°C by vigorous vortexing, and then centrifuged at 6,500 rpm for 15 min at 4°C. The supernatant was used to determine both the lipid peroxidation level and H_2_O_2_ concentration, as reported previously (Tsaniklidis et al., 2020). For each petal treatment, three biological replications were employed. The statistical analysis was performed by implementing a parametric one-way ANOVA, and the significance across treatments was deduced.

### RNA sequencing and data processing

To investigate the transcriptomic dynamics of the two cultivars upon *M. laxa* challenge in detached petal disk assays, two time series were employed, reflecting early pear responses upon infection. Total RNA extraction from each of the 24 samples (three biological replicates per treatment) was performed using the Monarch Total RNA Miniprep Kit (NEB, Europe). RNA integrity was assessed using the RNA Nano 6000 Assay Kit of the Bioanalyzer 2100 system (Agilent Technologies, CA, USA), and 500 ng of total RNA was used for sequencing libraries using the PT042 NGS RNA Library Prep Set (Novogene Ltd, Cambridge, UK). The library products were sequenced on the Illumina NovaSeq 6000 platform, and 150 bp paired-end reads were generated. Raw data were cleaned using cutadapt (v3.0) (Martin, 2011), and reads were mapped against the *Pyrus communis* (cv. Bartlett) reference genome available at GDR (Genome Database for Rosaceae) (Linsmith et al., 2019) using HISAT2 (Mortazavi et al., 2008). Mapped reads were filtered, sorted, and indexed using Samtools (Danecek et al., 2021), and gene counts were retrieved for each sample using HTSeq (Putri et al., 2022). Downstream data analysis was performed in R v.4.1.2 (R Core Team, 2022) on data normalized using DESeq2 (Love et al., 2014).

### DEG identification in response to *Monilinia laxa* infection and their functional enrichment

To identify DEGs using DESeq2 in response to *M. laxa* infection at both cultivars, all eight treatments were allocated to four comparison groups, namely, K2, K48, S2, and S48, based on dual comparisons among the inoculated (ML) and control (CT) treatments for each time point and cultivar. Transcripts with an absolute log2fold change value ≥2 and FDR-adjusted *p*-values <0.05 were considered as DEGs. Venn diagrams were created using the online tool at https://bioinfogp.cnb.csic.es/tools/venny/index.html. Gene ontology (GO) enrichment analysis of the DEGs was performed using the topGO R package (Alexa and Rahnenführer, 2009), and pathway enrichment analysis was conducted using the Kobas (v3.0) online tool (http://kobas.cbi.pku.edu.cn/), based on the Kyoto Encyclopedia of Genes and Genomes (KEGG) database (http://www.genome.ad.jp/kegg/).

### Weighted gene co-expression network analysis

WGCNA was performed using the RPKM values of the DEGs obtained from previously described pairwise comparisons. The correlation between genes was estimated using the Pearson correlation coefficient (PCC), which was used to calculate the distance matrix. WGCNA and calculations were performed using the WGCNA R package v1.70-3 (Langfelder and Horvath, 2008). The distance matrix was then used for dynamic hierarchical clustering and to build edges (connections) between nodes (genes) in the network.

The eight treatments were included in the WGCNA, and network topology research was executed from 1 to 20 soft thresholding powers using scale-free topology criteria and used a power of 9 to identify the co-expressed modules. The minimum module size was set to 30, and the merge cut height was set to 0.15 (to merge modules with at least 85% similarity). The correlations between one gene and all others were incorporated into an adjacency matrix, which was then transformed into a topological matrix (TOM) (Yip and Horvath, 2007). After hierarchical clustering, highly correlated genes were assigned to the same module (Ravasz et al., 2002).

After identifying the significant co-expression modules (ME ≥ 0.95), we performed a functional analysis to identify the biological functions and pathways involved in petal defense responses against *M. laxa* and then filtered the module eigengenes for the MM and GS absolute values ≥0.85. The eigengenes highly associated with the relatively tolerant cultivar infected with *M. laxa* were considered hub genes to construct regulatory networks. Regulatory network visualization and analysis of the highly connected genes were performed using Cytoscape v3.10.1 software (Shannon, 2003).

### Quantitative real-time PCR verification

RNA-seq data were validated using quantitative real-time PCR (qRT-PCR). First-strand cDNA was constructed using the LunaScript^®^ RT SuperMix Kit (NEB, Europe), and quantitative expression analysis was performed using the Luna^®^ Universal qPCR Master Mix (NEB, Europe) on a QuantStudio^®^ 5 Real-Time PCR System (Applied Biosystems, Europe). The expression profiles of nine randomly selected DEGs were analyzed by comparison with the actin-encoding reference gene (pycom15g30330). The relative gene expression log2 fold change of inoculated samples compared with controls was calculated according to the 2^−△△CT^ method (Livak and Schmittgen, 2001), using three technical replicates. The correlation between RNA-seq and qRT-PCR data was determined using a linear model. The gene-specific primers used are listed in **Supplementary Table S1**.

## Results

### *Monilinia laxa* infection on pear petals and flowers

**Figure 1:**
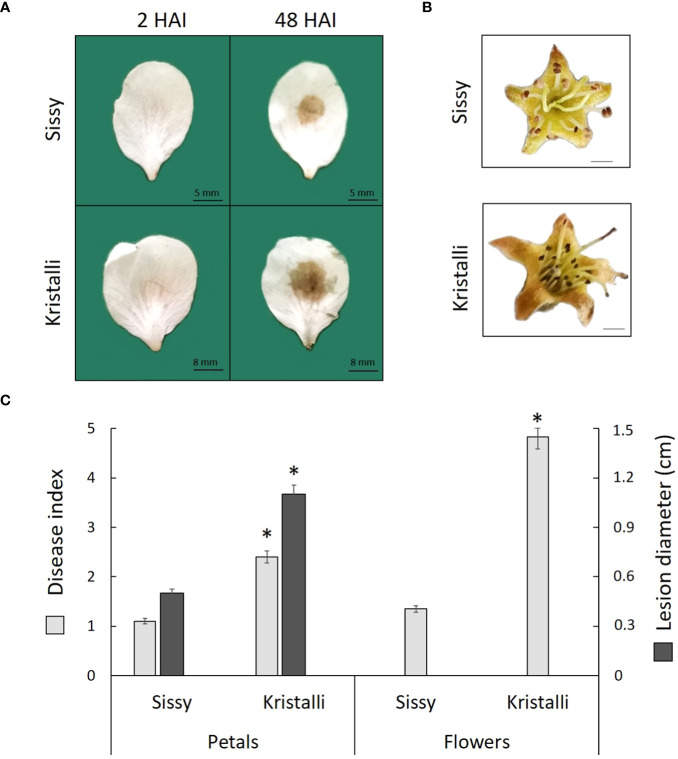
Disease symptoms of *Monilinia laxa* in the Kristalli and Sissy cultivars upon inoculation **(A)** in petals at 2 HAI and 48 HAI or **(B)** in flowers at 120 HAI. **(C)** Mean disease index values of both organs (48 HAI in petals and 120 HAI in flowers), along with mean values for necrotic lesion diameters in petals at 48 HAI upon *M. laxa* infection. Asterisks (*) indicate significant differences among the two cultivars according to the non-parametric Mann–Whitney *U*-test (*p* < 0.05) for disease index data and *t*-test for independent samples (*p* < 0.05) for the lesion diameter. Bars indicate the mean values of three biological replicates ± standard deviations.

All petals inoculated with *M. laxa* developed visual brown rot necrotic lesions around the inoculation sites at 48 HAI, while no symptoms were observed on the control samples mock inoculated with PDB. The disease index showed a higher disease severity in the sensitive Kristalli cultivar compared with the relatively tolerant Sissy cultivar (**Figure 1A**). Disease symptoms in the inoculated flowers showed also a differential disease severity among the two cultivars at 120 HAI (**Figure 1B**). The mean disease index values of the petals and flowers, along with the mean lesion diameters on petals, were significantly lower in the Sissy cultivar compared with the Kristalli cultivar (**Figure 1C**).

### Physiological changes of pear petals in response to *Monilinia laxa*

**Figure 2:**
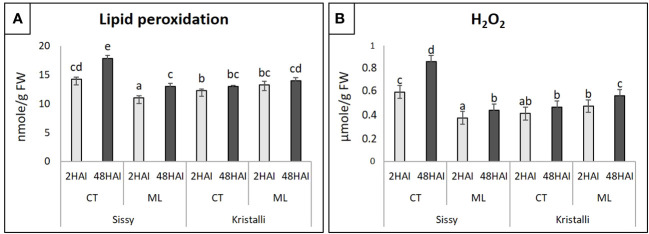
Physiological indicators of petal responses among mock-inoculated (CT) and infected petals (ML) of the pear cultivars Sissy and Kristalli across two time points after inoculation with *Monilinia laxa*. **(A)** Lipid peroxidation (thiobarbituric acid-reactive substances; TBARS). **(B)** Hydrogen peroxide (H_2_O_2_) levels. Bars indicate the mean values of three biological replicates ± standard deviations. Statistical analysis was performed using one-way ANOVA followed by Tukey’s multiple comparison *post-hoc* test (*p* < 0.05). Different letters represent statistically significant differences.

In the Sissy cultivar, both TBARS (lipid peroxidation) and H_2_O_2_ levels at S2CT and S48CT treatments were significantly higher than those of ML treatments, reaching their highest levels at 48 HAI (**Figures 2A, B**). In contrast, in the Kristalli cultivar, TBARS and H_2_O_2_ levels were significantly higher and progressively increased in the ML treatments compared with the respective K2CT and K48CT treatments. The comparison of inoculated petals (ML) from the two cultivars showed higher levels of both TBARS and H_2_O_2_ in the sensitive cultivar (Kristalli) (**Figures 2A, B**).

### RNA-seq analysis of pear petals after inoculation with *Monilinia laxa*

**Figure 3:**
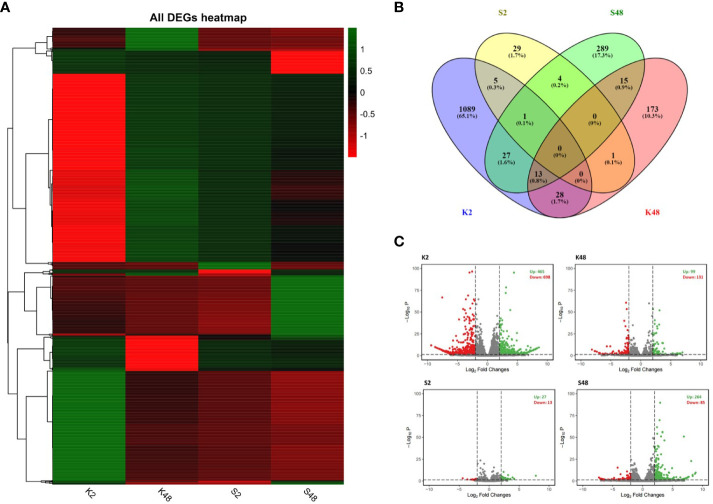
Summary of differentially expressed genes (DEGs) (|log2FC| > 2) across the four comparison groups (K2, K48, S2, S48). **(A)** Plot showing DEGs hierarchical clustering (green and red colors in each node of the dendrogram represent up- and downregulation of DEGs, respectively). **(B)** Venn diagram plots showing overlap of all DEGs and **(C)** volcano plots of the DEGs for each comparison group.

We constructed 24 sequencing libraries to study the changes occurring in the petals of both pear cultivars at the transcriptome level in response to *M. laxa* infection at two time points (2 HAI and 48 HAI). Clean reads were obtained (**Supplementary Table S2**) and mapped to the European pear genomic assembly of the cultivar “Bartlett” (referred to as BartlettDHv2.0). In total, 1,647 unique genes were identified as DEGs comparing inoculated and mock-inoculated petals of the two cultivars at 2 HAI and 48 HAI across the four comparison groups (K2, K48, S2, S48) (**Figure 3**). The lists of DEGs across the four comparison groups are shown in **Supplementary Tables S3**-**S6**. The expression patterns of our RNA-seq data suggest a dynamic, cultivar-specific, and time-dependent transcriptional reprogramming upon *M. laxa* inoculation on petals across both cultivars (**Figure 3A**). Notably, no common DEGs were detected among the four comparison groups, whereas 65.1% of the DEGs were identified exclusively in the K2 comparison group (**Figure 3B**). Furthermore, the highest number of DEGs was identified in the K2 group (1,163 in their number) and the lowest number was in the S2 group (40 in their number). The proportion of up-/downregulated DEGs was 0.663, 0.755, 2.076, and 3.105 for the four comparison groups K2, K48, S2, and S48, respectively (**Figure 3C**). It is clearly evident that in both time points after *M. laxa* infection, the proportion of up-/downregulated DEGs was higher in the Sissy compared with the Kristalli cultivar.

### Functional annotations and classifications of DEGs

**Figure 4:**
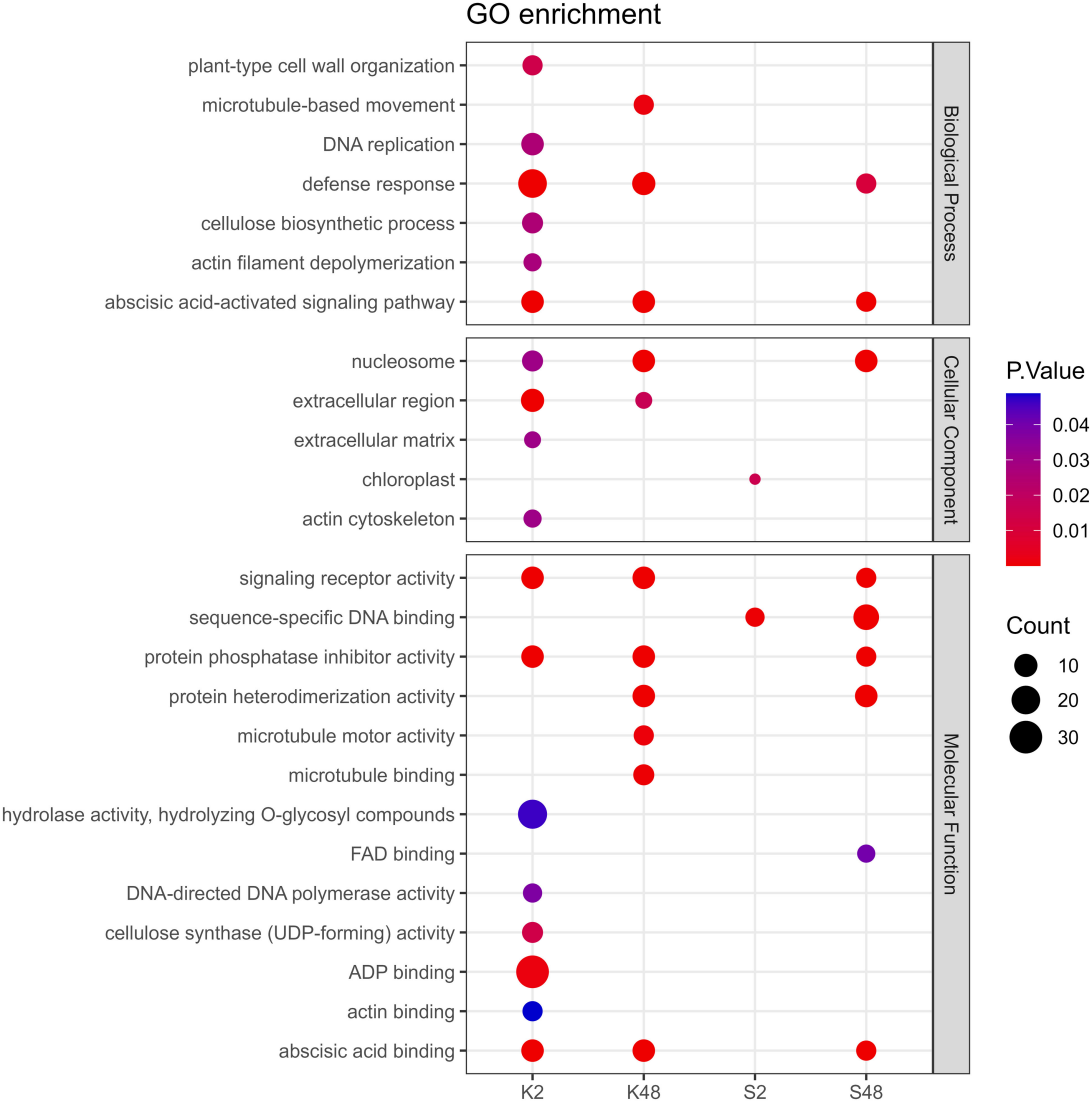
Dot plot showing the GO enriched terms of DEGs identified across the four comparison groups (K2, K48, S2, S48) related to the biological process (BP), cellular component (CC), and molecular function (MF).

**Figure 5:**
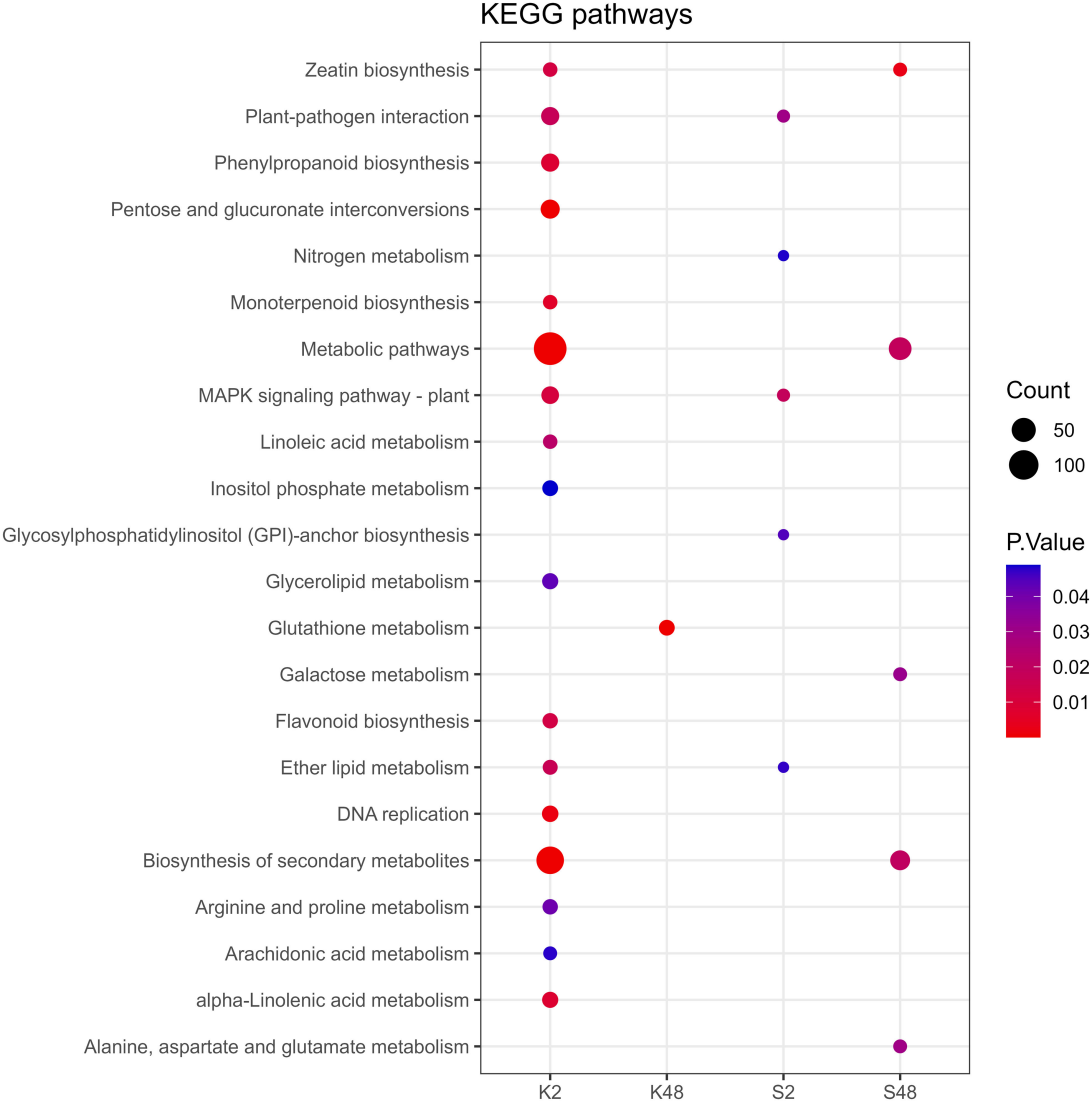
Dot plot showing the KEGG enriched pathways of DEGs identified across the four comparison groups (K2, K48, S2, S48).

Following GO term enrichment analyses, DEGs were allocated to significant functional annotations and categorized based on their molecular function (MF), cellular component (CC), and biological process (BP) (**Figure 4**, **Supplementary Tables S3**-**S6**). In the Kristalli cultivar, the GO term “defense response” was enriched mainly at 2 HAI, whereas in the Sissy cultivar, it was exclusively at 48 HAI. Among the enriched GO terms related to molecular functions, the terms “sequence-specific DNA binding” and “FAD binding” were recorded only in the Sissy cultivar, particularly at 48 HAI (**Figure 4**). A KEGG functional enrichment analysis was also conducted at each time point to further examine the metabolic pathways and biological functions of DEGs in both cultivars upon *M. laxa* infection in the petals. Pathways related to “zeatin biosynthesis” and “biosynthesis of secondary metabolites” were enriched at 2 HAI and 48 HAI for the Kristalli and Sissy cultivars, respectively (**Figure 5**). The “galactose metabolism” pathway was exclusively enriched in the Sissy cultivar at 48 HAI, while the “flavonoid biosynthesis” pathway along with pathways related to linolenic metabolism was evident only at 2 HAI in the Kristalli cultivar. As expected, both “plant–pathogen interaction” and “MAPK signaling” pathways were enriched in both cultivars at 2 HAI (**Figure 5**, **Supplementary Tables S3**-**S6**).

### Transcriptional changes in the Kristalli cultivar upon *Monilinia laxa* inoculation

**Figure 6:**
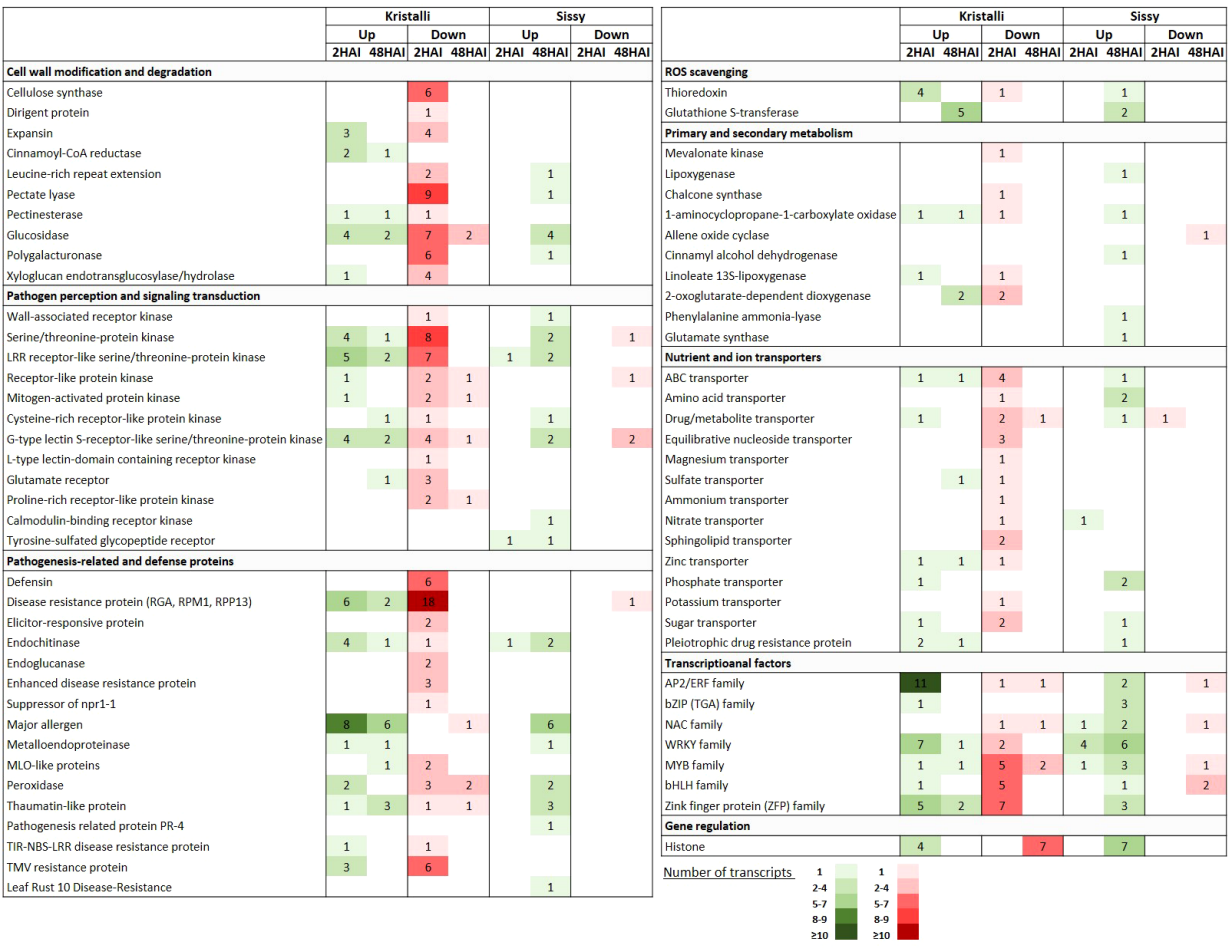
Selection of key DEGs upregulated (Up) and downregulated (Down) in pear petals of both cultivars after inoculation with *Monilinia laxa* at 2 HAI and 48 HAI. For each gene category, the numbers of the differentially expressed transcripts are shown.

A large number of key DEGs involved in cell wall modification and degradation processes were downregulated at 2 HAI, including genes encoding cellulose synthase (CesA), extensin containing leucine-rich repeat (LRR-EXT), expansin (EXP), pectate lyase (PL), polygalacturonase (PG), glucosidase (GL), and xyloglucan endotransglucosylase/hydrolase (XTH), along with a dirigent protein gene. In contrast, fewer DEGs were upregulated at this time point, including two cinnamoyl-CoA reductase (CCR), three EXP, and four GL-encoding genes. This transcriptional reprogramming was less evident at 48 HAI (**Figure 6**, **Supplementary Table S7**).

Pattern recognition receptors (PRRs) were significantly induced upon infection mainly at 2 HAI, including various types of membrane-associated receptor-like kinases (RLKs) or receptor-like proteins (RLPs). Most of these DEGs encode RLKs possessing serine/threonine kinase activity (SRKs) and LRR domains (LRR-RLKs) or encode receptors with lectin domains (LecRKs). However, DEGs involved in signal transduction at 2 HAI were mainly downregulated, particularly STKs (serine/threonine-protein kinases) encoding DEGs. Among the downregulated DEGs involved in pathogen perception at 2 HAI were a WAK (wall-associated kinase), three GLR (glutamate receptor-like), and two PERK (proline-rich receptor-like kinase)-encoding genes. At 48 HAI, the numbers of up- and downregulated DEGs involved in pathogen perception and signal transduction were quite similar (**Figure 6**, **Supplementary Table S8**).

Our RNA-seq data indicated that DEGs encoding pathogenesis-related and defense proteins were mainly downregulated, particularly at 2 HAI. Thus, numerous DEGs encoding disease resistance genes (*RGA*, *RPM1*, *RPP13*), defensins, elicitor-responsive, enhanced disease resistance, and TMV resistance proteins were significantly suppressed. However, a few DEGs encoding endochitinase were upregulated at 2 HAI along with other gene members from the PR-10 family, such as those encoding major allergens. This trend was slightly inverted at 48 HAI with a higher number of upregulated defense-related DEGs than those that were suppressed (**Figure 6**, **Supplementary Table S9**).

Several TF-encoding genes belonging to the AP2/ERF and WRKY families were mainly upregulated at the early time point. In contrast, members of MYBs and bHLHs were downregulated at an early time point. A few TFs were induced at 48 HAI with similar expression patterns (**Figure 6**, **Supplementary Table S10**). Furthermore, DEGs involved in redox homeostasis and scavenging, such as thioredoxin (TXN) and glutathione S-transferase (GST)-encoding genes, were upregulated at 2 HAI and 48 HAI, respectively. In contrast, DEGs involved in secondary metabolism were mostly induced at an early time point and were mostly suppressed (**Figure 6**, **Supplementary Table S11**), such as those encoding nutrient and ion transporters (**Figure 6**, **Supplementary Table S12**). Finally, seven histone-encoding DEGs were constitutively suppressed at 48 HAI, whereas four of them were upregulated at 2 HAI (**Figure 6**).

### Transcriptional changes in the Sissy cultivar upon *Monilinia laxa* inoculation

A less abundant repertoire of transcriptional responses related to cell wall modification and degradation processes was observed in the cv. Sissy (**Figure 6**, **Supplementary Table S7**). Thus, only four *GLs* were upregulated at 48 HAI, along with three DEGs encoding members of the *LRR-EXT*, *PL*, and *PG* genes. This pattern was also retained in the DEGs related to pathogen perception and signal transduction. Thus, among the upregulated DEGs at the later time point (48 HAI) were a *WAK*, two *STKs*, two *LRR-RLKs*, and two *G-type LecRKs*. In addition, two genes encoding cysteine*-*rich receptor*-*like kinase (CRK) and calmodulin-binding receptor-like cytoplasmic kinase (CRCK), both of which belong to the receptor-interacting protein kinase (RIPK) family, were also upregulated at 48 HAI. A less abundant inventory of *PRR* genes was observed at 2 HAI (**Figure 6**, **Supplementary Table S8**).

Various types of DEGs encoding pathogenesis-related and defense proteins were also constitutively upregulated at the later time point, whereas six members of *major allergens* genes belonging to the PR-10 family were induced. Notably, only one well-known disease-resistance gene (pycom02g24320) was suppressed at this time point. In contrast, only one endochitinase encoding DEG (pycom04g04190) was upregulated at 2 HAI (**Figure 6**, **Supplementary Table S9**).

In contrast to the cv. Kristalli, numerous TF-encoding genes belonging to various families, such as *WRKYs*, *bZIPs*, *MUBs*, and *ZFPs*, were mostly upregulated at 48 HAI (**Figure 6**, **Supplementary Table S10**). In particular, four and six *WRKY*s were constitutively upregulated at 2 HAI and 48 HAI, respectively. Furthermore, at the later time point, DEGs involved in redox homeostasis and scavenging (*TXN*, *GSTs*), as well as in the induction of secondary and primary metabolism, such as homologs of *lipoxygenases* (*LOXs*), *cinnamyl alcohol dehydrogenases* (*CADs*), *phenylalanine ammonia-lyases* (*PALs*), and *glutamate synthase* (*Glts*) genes, were all upregulated (**Figure 6**, **Supplementary Table S11**). The same expression pattern was also evident for a few transporter-encoding DEGs (**Figure 6**, **Supplementary Table S12**). Notably, seven histone-encoding DEGs were constitutively upregulated at 2 HAI (**Figure 6**).

### Weighted gene co-expression network analysis

**Figure 7:**
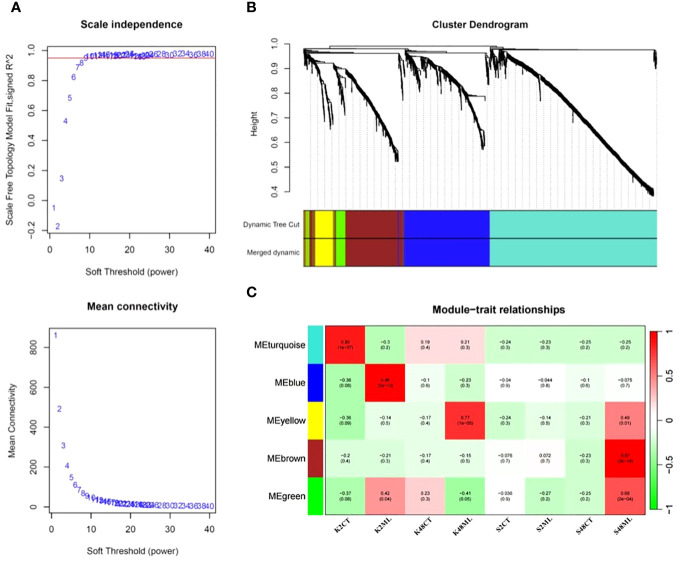
Scale independence and mean connectivity **(A)**, cluster dendrogram **(B)**, and module–trait relationship **(C)** were obtained through the WGCNA using the 1,647 DEGs identified in pear petals across the eight treatments.

To identify co-expression modules and hub genes involved in the transcriptional regulatory networks governing immunity responses and tolerance of pear petals to *M. laxa*, we conducted a WGCNA, including the 1,647 DEGs identified in the four comparison groups. Our results revealed five co-expressed modules, namely, turquoise, blue, yellow, brown, and green, with 789, 412, 89, 316, and 66 co-expressed genes, respectively (**Figure 7**).

Analysis of the interaction between the co-expression and the eight sample treatment modules revealed the expression levels of eigengenes (idealized representative genes) within each module (**Figure 7C**). The results indicated that the brown module (318 genes, ME = 0.97, *p* = 3 × 10^−14^) exhibited the highest correlation with *M. laxa* infection in the S48ML treatment, suggesting that the DEGs belonging to this module may play significant roles in the relative tolerant cultivar (Sissy) during *M. laxa* infection compared with the Kristalli cultivar (**Figure 7C**). In contrast, the blue module was significantly correlated with the sensitive cultivar at 2 HAI. In our case study, we focused only on the brown module because we aimed to detect key genes regulating the tolerance of pear petals to *M. laxa*.

### *Monilinia laxa* tolerance-related module analysis and identification of hub genes in the Sissy cultivar

The brown module, a unique co-expression module, was highly correlated with *M. laxa* infection in the relevant tolerant cultivar (Sissy) at 48 HAI and grouped 318 genes that were involved in defense response, plant-type cell wall organization, and cellulose biosynthesis for the biological process GO terms, in the extracellular matrix and nucleosome for the cellular component GO terms, and in FAD and DNA binding for the molecular function GO terms. The KEGG pathway analysis revealed that the brown module genes were involved in MAPK signaling and metabolic pathways, biosynthesis of secondary metabolites (monoterpenoids and zeatin biosynthesis), and plant–pathogen interactions (**Supplementary Table S13**).

**Figure 8:**
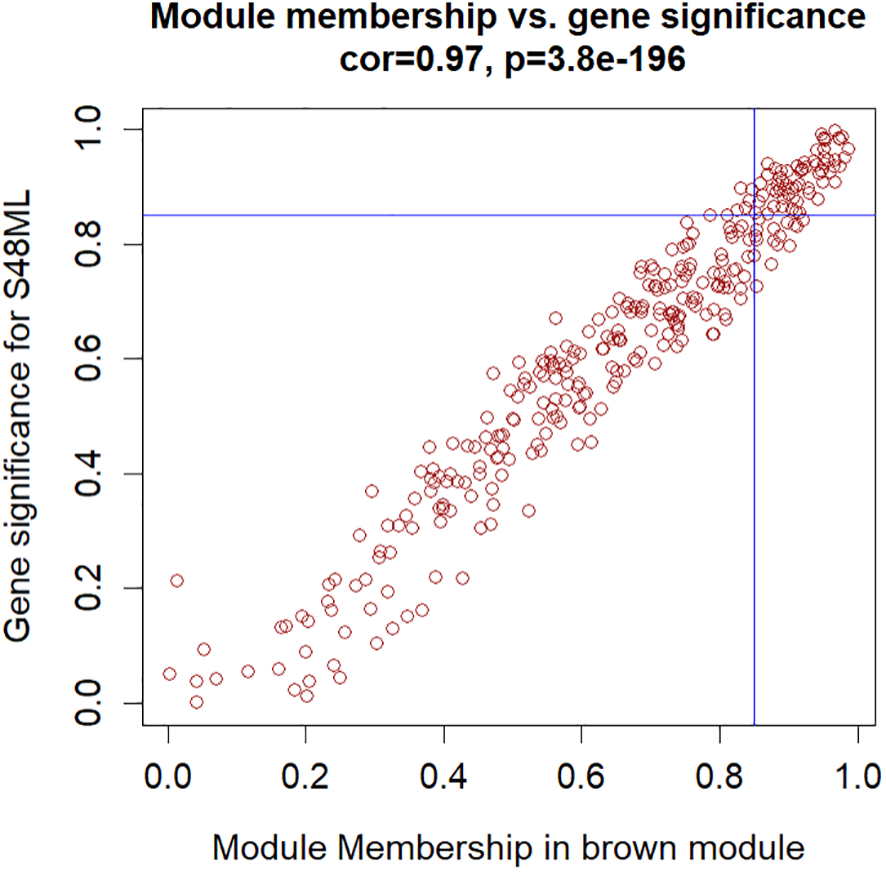
Hub genes in the brown module selected based on the module membership (MM) vs. gene trait significance (GS) for the S48ML treatment.

The hub genes were further selected among the genes involved in these biological functions to meet the absolute value of MM ≥0.85 and GS for S48ML ≥0.85 (**Figure 8**, **Supplementary Tables S14**, **S15**). Six genes were identified: pycom08g05900 (cytokinin dehydrogenase 7), pycom05g27470 (WRKY transcription factor 65), pycom10g22220 (WRKY transcription factor 71), pycom15g24670 (heat shock transcription factor B1a, HSF-B1a), pycom15g32240 (transcription factor TGA3-like), and pycom17g13130 (WRKY transcription factor 28).

Functional analysis of the hub genes revealed their significant involvement in FAD- and DNA-binding molecular function GO terms and in the zeatin biosynthesis KEGG pathway, suggesting their relevant role in modulating gene expression patterns of petal immunity responses in the cv. Sissy toward induced tolerance in response to *M. laxa* infection. Thus, the six hub genes were used as bait genes to construct a brown module regulatory network.

**Figure 9:**
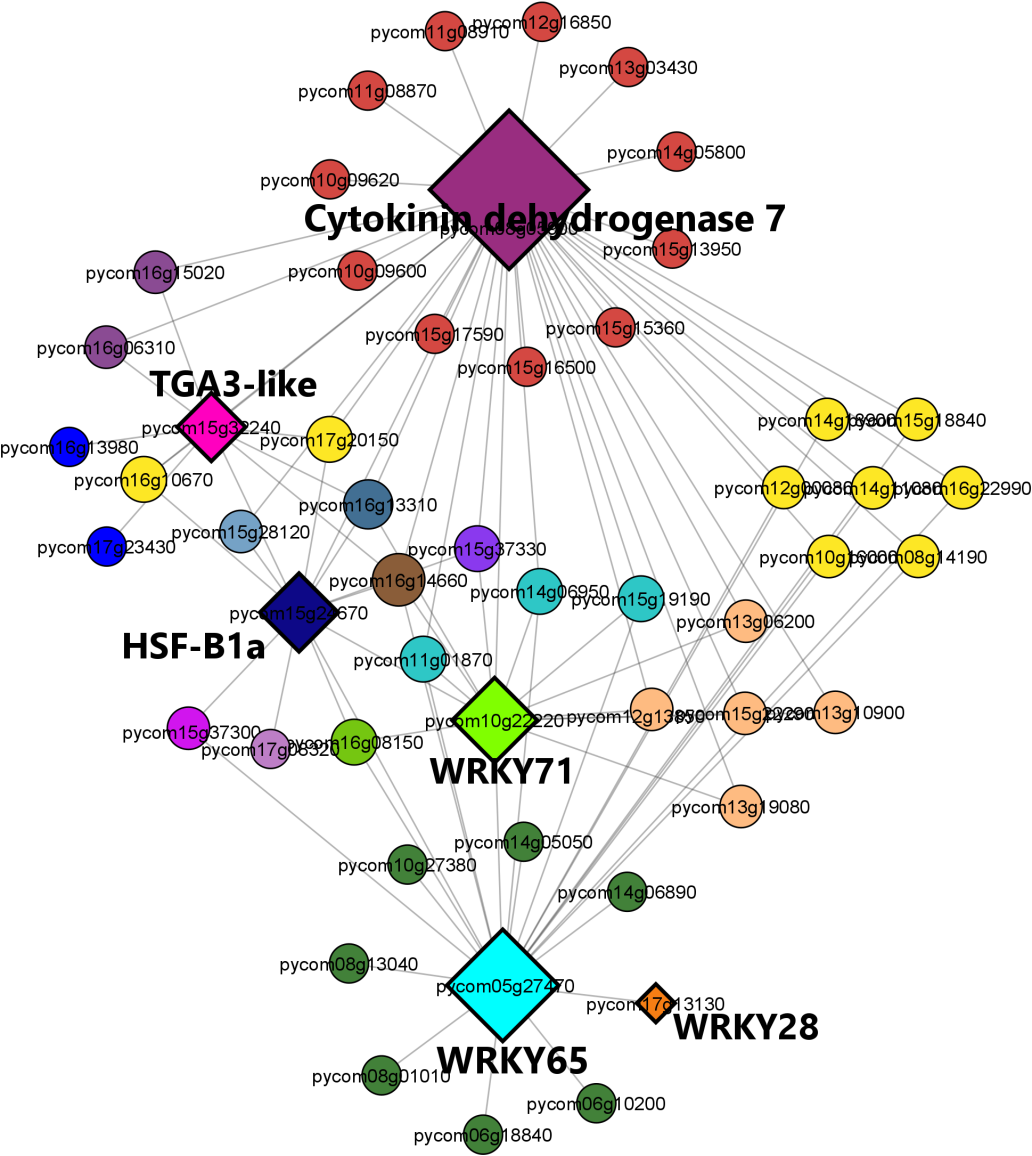
Regulatory network visualization of the brown module correlated with the Sissy cultivar at 48 HAI. The hub genes are diamond-shaped and their annotations are bold-highlighted in the network.

**Figure 10:**
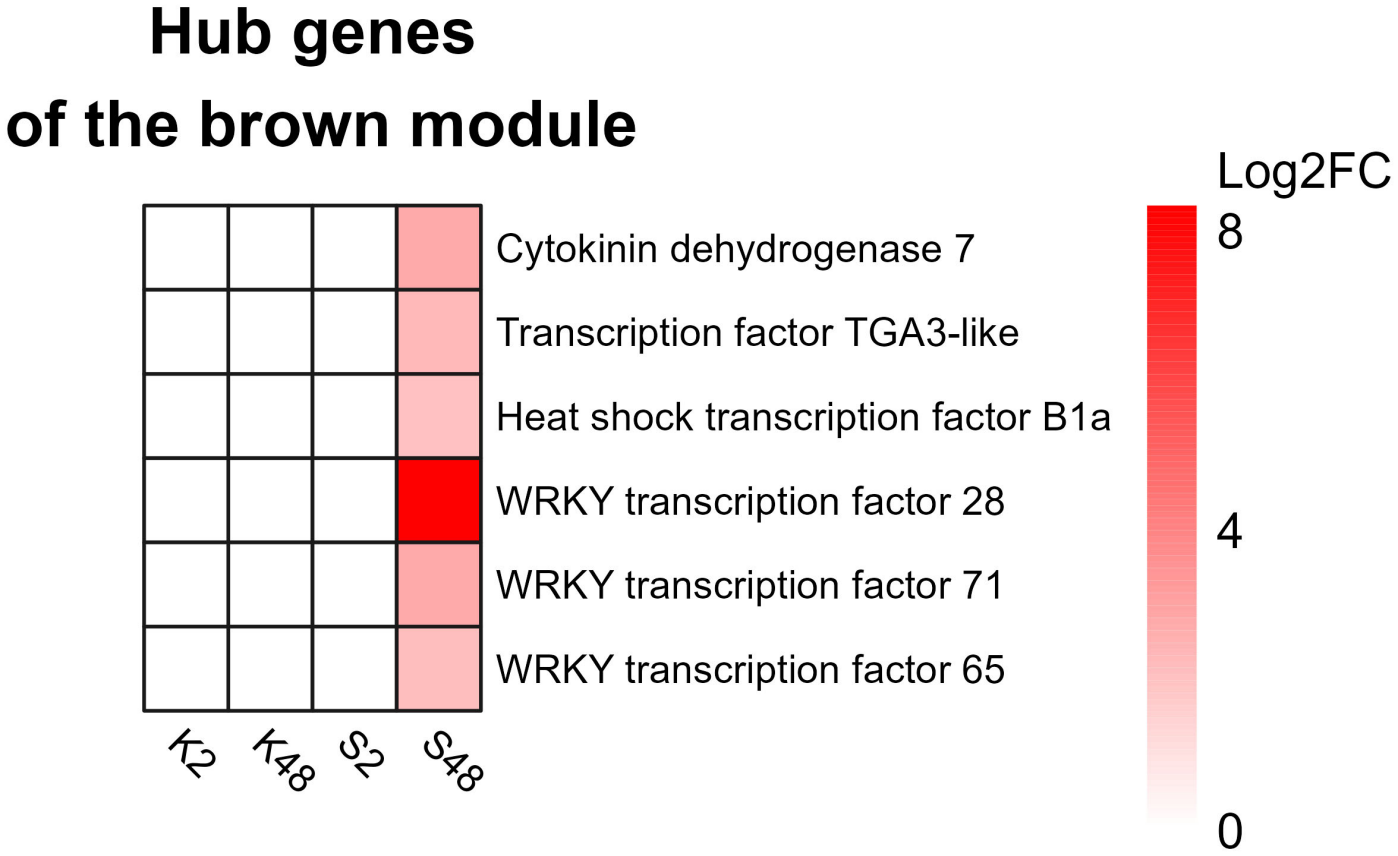
Heatmap showing the expression patterns of the hub genes potentially involved in the relative tolerance of the Sissy cultivar to *Monilinia laxa*.

The brown module regulatory network (**Figure 9**) revealed the presence of 52 nodes connected with 91 edges. The most connected genes (diamond-shaped hub genes) within the network encoded the cytokinin dehydrogenase 7 gene, followed by WRKY 65, WRKY 71, heat shock factor HSF-B1a, TGA3-like, and WRKY28 TFs. Finally, the expression patterns of the key genes identified for *M. laxa* tolerance in the brown module for Sissy petals at 4 8HAI are graphically presented in the heatmap (**Figure 10**, **Supplementary Table S16**).

### Validation of RNA-seq data using qRT-PCR

A linear model was used to model the correlation between RNA-seq and qRT-PCR data (log2 fold change of nine randomly selected genes) for the K2 and S48 comparison groups (**Supplementary Table S17**, **Supplementary Figure 1**). The goodness of fit was determined by the coefficients of determination (*R* ^2^) which were equal to 0.82 and 0.89 for the K2 and S48 groups, respectively, indicating a good fit between RNA-seq and qRT-PCR data.

## Discussion

In pear petals, the transcriptional responses to infection with *M. laxa* remain largely unknown. Thus, the overarching objective of this study was to decipher the transcriptome dynamics and regulatory mechanisms involved in the early stages of infection with *M. laxa* in the petals of two pear cultivars with differences in their sensitivity to fungal diseases. Our results suggest that, during these compatible interactions, both cultivars initiate a basal defense response to some extent, which is quite distinctive at different stages of the infection. However, in both cultivars, the specific time-dependent transcriptional reprogramming upon infection was accompanied by a failure to restrict fungal growth and disease progression, which was more pronounced in the susceptible cultivar Kristalli.

In both cultivars, the expression patterns of our RNA-seq data in petals suggest a dynamic reprogramming upon infection, as observed in other studies, such as in strawberry, grapevine, and rose petals infected by *B. cinerea* (Haile et al., 2017; Liu et al., 2018; Xiao et al., 2022). Notably, DEGs involved in plant–pathogen interaction, immune signal transduction, biosynthesis of secondary and primary metabolism, and other defense-related responses were induced in delay in the Sissy cultivar, which is relatively tolerant to *M. laxa* compared with the Kristalli cultivar. The expression profiles of the DEGs were also significantly different among the cultivars. Thus, in the Kristalli cultivar, the majority of DEGs were suppressed at 2 HAI, while in the Sissy cultivar, DEGs were mostly upregulated at 48 HAI. In contrast, a less abundant inventory of key genes and pathways involved in defense responses was observed at 48 HAI in the Kristalli cultivar. Significant downregulation of a high proportion of DEGs in the Kristalli cultivar is consistent with other studies on hosts infected with *M. laxa* (Balsells-Llauradó et al., 2020), as well as those infected with *B. cinerea* (Petrasch et al., 2019; Zambounis et al., 2020). This downregulated expression in Kristalli at 2 HAI was mainly attributed to the significant and rapid downregulation of specific DEGs and pathways associated with defense responses, biosynthesis of secondary metabolites, and plant–pathogen interactions. Notably, these pathways are suppressed during early responses in both compatible and incompatible interactions with *B. cinerea* (Smith et al., 2014; Kong et al., 2015; Petrasch et al., 2019).

The cell wall constitutes a structural barrier in response to pathogen attack (Haile et al., 2019; Wang et al., 2020b). At the same time, it is also the initial target for *M. laxa* during penetration as its disassembly contributes to susceptibility to pathogen invasion (Garcia-Benitez et al., 2019; Haile et al., 2019). Particularly, in the Kristalli cultivar, many DEGs involved in cell wall modification were differentially regulated at 2 HAI, as already described in strawberries upon *Botrytis* infection (Haile et al., 2019; Xiao et al., 2022). Thus, the constitutive upregulation of DEGs associated with hampering pathogen penetration, including *CCR* genes, along with the simultaneous suppression of *PG* genes, strongly mitigated the effect of susceptibility during *M. laxa* infection, as was also reported during *B. cinerea* infection in kiwi fruits (Cantu et al., 2008; Zambounis et al., 2020). *Monilinia laxa* may further stimulate petal softening by manipulating *PL* genes during the early infection stage, as has been reported in *Botrytis* (Lu et al., 2019). Indeed, in our study, we found nine *PL* genes that were downregulated in the K2 comparison group, implying their involvement in the induction of early defense responses. Previously, the suppression of the *PL* gene was reported to reduce susceptibility to *B. cinerea* and increase the concentration of cellulose and hemicellulose in tomatoes (Yang et al., 2017). Furthermore, *Gl* genes that degrade cellulose and hemicellulose (Blanco-Ulate et al., 2013) were mainly suppressed in the K2 group, indicating that they might partially contribute to the deployment of defense responses to some extent against *M. laxa* infection. On the other hand, in the same comparison group, the downregulation of *CesA* and *LRR-EXT* genes that influence cell wall extensibility and susceptibility (AbuQamar, 2014) might have facilitated pathogen colonization, along with the suppression of four *XTH* genes, which may be related to the thickening of cell walls (Liu et al., 2018; Wang et al., 2020b). The downregulation of a *dirigent* protein gene involved in the biosynthesis of lignans, which was previously found to be the most upregulated DEG in unripe strawberry fruits during cell wall reinforcement upon *Botrytis* elicitation (Haile et al., 2019), is another potential susceptibility-related response in the Kristalli cultivar that could be taken into consideration at the early infection stage. Overall, we are tempted to speculate that transcriptional changes in DEGs related to cell wall degradation and modification processes might contribute to Kristalli sensitivity to *M. laxa*, whereas the relative tolerance of the Sissy cultivar might not be regulated by such DEGs, at least at the early time point upon infection.

The high induction of DEGs involved in pathogen perception and signal transduction, particularly in Kristalli, highlights the important role of PTI in the pear–*M. laxa* pathosystem. Thus, in Kristalli, PTI seems to be mediated through the induction of an array of *PRR* genes, such *as LRR*-*RLKs* and *LecRKs*, which were however mainly suppressed at the early time point. Notably, *LRR-RLK* genes in the Sissy cultivar were constitutively upregulated at both time points, suggesting their putative role in higher innate immunity response. Several *RLK* genes have been previously shown to induce immune responses in necrotrophs (De Cremer et al., 2013; Zambounis et al., 2020). Furthermore, *LecRKs* are also involved in the defense responses of rose petals against *Botrytis* (Liu et al., 2018). It is worth mentioning that among the induced RLK genes, the G-type LecRKs are known to be involved in plant defense (Lannoo and Van Damme, 2014). In addition, in the S48 comparison group, the upregulation of a *WAK* receptor gene might provide further evidence of its involvement in *M. laxa* recognition, whereas another *WAK* gene, along with *GLRs* and *PERKs*, was suppressed in the K2 group. Previously, it was reported that during infection with *Botrytis*, *WAK* receptors were also upregulated in ripe strawberry fruits, lettuce, and rose petals (De Cremer et al., 2013; Liu et al., 2018; Haile et al., 2019). Our RNA-seq data also revealed that in S48, in contrast to the K48 group, various classes of branched kinases (STKs, CRKs, and CRCK) known to be involved in immune signaling pathways (Tang et al., 2017) were upregulated. Among them, STKs and CRKs are well-known plant defense regulators (Zambounis et al., 2020) and were similarly induced in lettuce after *B. cinerea* infection at 48 HAI (De Cremer et al., 2013).

Reprogramming of both secondary and primary metabolism putatively activates host defense responses (Agudelo-Romero et al., 2015). The upregulation of the *GltS* gene in the cv. Sissy at 48 HAI seems to further promote its tolerance, as it is known that glutamate triggers long-distance calcium-based plant defense signaling (Toyota et al., 2018). Secondary metabolites actively participate in defense pathways to tackle invading pathogens (Zaynab et al., 2018). Indeed, in the Kristalli cultivar, the biosynthesis of secondary metabolites was selectively activated in delay to some extent, which is consistent with previous studies (Agudelo-Romero et al., 2015; Xiong et al., 2018; Haile et al., 2019). These delayed defense responses in Kristalli were also supported by the highly enriched KEGG terms of “metabolic pathways” and “biosynthesis of secondary metabolites.” These results are consistent with previous findings in a tomato genotype susceptible to *Botrytis*, where the induction of defense responses and particularly the metabolic shunt for the biosynthesis of secondary metabolites were not observed until 48 HAI (Smith et al., 2014). It is known that JA has important regulating roles associated with defense responses against necrotrophic fungi (Blanco-Ulate et al., 2013; Sham et al., 2014; Agudelo-Romero et al., 2015; Haile et al., 2019). In our study, JA was also highlighted as the principal phytohormone in signaling transduction-mediated responses in Sissy based on the S48 group, as a *lipoxygenase* gene, which is related to JA biosynthesis, and was upregulated at 48 HAI. In this line, the activation of a *CAD* gene might also contribute to the elevated tolerance of the Sissy cultivar, as such genes are involved in lignin biosynthesis and resistance to pathogens (Li et al., 2022). In addition, one PAL*-*encoding gene, the key enzyme in the phenol biosynthesis pathway, was found upregulated in the S48 group, whereas its triggering is likely correlated with the Sissy higher competence to block *M. laxa* growth. In Kristalli, genes encoding 2-oxoglutarate-dependent dioxygenase, which is also involved in JA biosynthesis, were suppressed at 2 HAI and upregulated at 48 HAI. Notably, in the K2 group, the pathway of “alpha-linolenic acid metabolism” was enriched through the induction of linoleate 13S-lipoxygenase-encoding genes, suggesting a role of this pathway in the activation to some extent of primary biosynthetic pathways. Finally, we are tempted to speculate that although induction of the phenylpropanoid biosynthesis pathway was recorded in both cultivars, it was insufficient to restrict fungal growth and disease progression, as previously reported in ripe grapes after infection with *B. cinerea* (Agudelo-Romero et al., 2015).

Upon challenge with necrotrophic phytopathogens, a large set of TF families play important roles in the orchestration and regulation of defense mechanisms (Smith et al., 2014; Tsuda and Somssich, 2015; Liu et al., 2018; Haile et al., 2019). It is also possible that some of these TFs are involved in susceptibility (Agudelo-Romero et al., 2015). In our study, the transcriptional reprogramming of several TF-encoding DEGs was revealed by a quite different and time-dependent expression pattern between the two cultivars. Thus, in the K2 group, several TF-encoding genes belonging to the AP2/ERF family were mainly upregulated at the early time point. Considering that *ERFs* are responsive genes in ethylene biosynthesis and act as susceptibility factors upon challenging with pathogens (Agudelo-Romero et al., 2015; Petrasch et al., 2019), *M. laxa* may rapidly manipulate their induction in Kristalli, thereby accelerating further its susceptibility to *M. laxa.* Members of the bHLH family are also characterized as JA-mediated transcriptional regulators that act cooperatively with other TFs (Goossens et al., 2017), such as MYBs, in plant defense responses against pathogen attacks (Vailleau et al., 2002). The downregulation of MYBs and bHLHs in the K2 group further indicates the suppression of Kristalli defense mechanisms, enhancing its high susceptibility. In contrast, ZFPs and MUBs, which regulate overlapping signaling pathways and metabolic modulation toward the establishment of disease resistance, were among the most abundantly induced TFs in the S48 group.

A virulence factor exploited by *M. laxa* to manipulate host responses and facilitate colonization is the production of ROS, which leads to an oxidative burst that orchestrates the hypersensitive response (HR) and promotes susceptibility (Balsells-Llauradó et al., 2020). In our study, physiological indexes of pear petals among the two cultivars upon ML treatment showed higher levels of both TBARS and H_2_O_2_ in the sensitive cultivar than in the relatively tolerant cultivar Sissy. As no induction of any *Rboh* (*respiratory burst oxidase homolog*) genes that play an important role in redox homeostasis was observed in either cultivar, the scavenging strategy seems to be mediated by the induction of *TXN* and *GST* genes that directly participate in the ROS-scavenging pathway (Zheng et al., 2020), which were mainly upregulated in the Kristalli cultivar at both time points.

The activation of *PRs* and defense-related DEGs in the S48 group further indicates a delay in the immune responses. These responses were also evident to a lesser extent at the later time point in Kristalli, whereas at 2 HAI, such DEGs were primarily suppressed in the susceptible cultivar. However, in both cultivars, DEGs encoding homologs of major allergen proteins belonging to the PR-10 group family were constitutively upregulated at 48 HAI, and also in the K2 group, suggesting an enhancement of the JA-mediated transduction of defense signaling (Casañal et al., 2013). These genes were also induced in strawberry and kiwi fruits challenged with *B. cinerea* (Xiong et al., 2018; Zambounis et al., 2020). Similar expression patterns were recorded for another class of the PR-10 family encoding metalloendoproteinase (MMP) genes that play pivotal regulatory roles in homeostasis during PTI (Zhao et al., 2017), while a specific MMP protein is required for disease resistance against *B. cinerea* in tomato (Li et al., 2015). However, in Kristalli, the downregulation at the early time point of numerous disease resistance genes, such as those encoding defensin, elicitor-responsive, enhanced disease resistance, and TMV resistance proteins, further highlights the suppression of any defense-related responses promoting the susceptibility of this cultivar. However, in Sissy, all except one disease-related gene were upregulated at 48 HAI such as those encoding thaumatin, endochitinase, and peroxidase proteins.

At both stages of infection for the Sissy and Kristalli cultivars, respectively, an abundant number of DEGs encoding nutrients and ion transporters were either upregulated or downregulated. Several of these might have been utilized by the pathogen to obtain nutrients for its own needs from decayed host cells (Xiong et al., 2018). Among the upregulated DEGs encoding ABC transporters at 48 HAI, members of the G family are involved in the transport and secretion of secondary metabolites in plants challenged by pathogens, whereas fungi have developed similar transporters to export it from their cytosol (Khare et al., 2017).

Co-expression network analysis has become a powerful tool that is commonly used for the identification or prediction of new genes and TFs associated with crop pathogen resistance (Amrine et al., 2015; Liu et al., 2019; Cui et al., 2022; Kumar et al., 2022). Here, WGCNA was used to identify co-expression modules and hub genes correlated with *M. laxa* tolerance in pear petals. The analysis classified the pathogen-responsive genes of both cultivars into five co-expression modules. The most significant module (brown module) was positively correlated to the Sissy cultivar at 48 HAI (S48ML treatment). The functional analysis revealed that genes within this module were mainly involved in DNA- and FAD-binding molecular functions, which further supports the hypothesis that this co-expression module is involved in the transcriptional regulation of the Sissy cultivar in response to *M. laxa*. Five TFs and cytokinin dehydrogenase-encoding genes were identified as hubs in this module regulatory network.

Among the TF hubs, three belonged to the WRKY family: *WRKY28*, *WRKY65*, and *WRKY71*. A study conducted on the *Arabidopsis thaliana* and *B. cinerea* pathosystem revealed that overexpression of At*WRKY28* led to *A. thaliana* disease resistance through the positive regulation of JA and ET biosynthesis-related genes (Wu et al., 2011). In pear petals, *WRKY28* was highly upregulated (log2FC = 8.33) in the S48 comparison group compared with the other TFs. Furthermore, *WRKY65* and *WRKY71* were upregulated only in this comparison group (S48). These genes are known to be involved in plant resistance to fungal and bacterial pathogens. The silencing of *PlWRKY65* from *Paeonia lactiflora* induced a higher sensitivity of the mutant plants to *Alternaria tenuissima* infection (Wang et al., 2020a), while overexpression of maize *ZmWRKY65* in *Arabidopsis* transgenic plants enhanced their resistance to *B. cinerea* and *Pseudomonas syringae* pv*. tomato* DC3000 (Pst) infection via the activation of *PR* genes (Huo et al., 2021). Both *WRKY65* orthologs exerted a regulatory effect on disease hormone signaling pathways, resulting in a more resistant phenotype. The *WRKY71 TF* was also a hub gene and had the same expression pattern as previous *TFs* in the Sissy cultivar. Overexpression of such a *WRKY* gene in rice (*OsWRKY71*) also resulted in improved resistance to *Xanthomonas oryzae* pv*. oryzae* (Liu et al., 2007).

DEGs encoding TGA3-like and HSF-B1a TFs were also identified as hub genes in the regulatory network which correlated with the relatively tolerant cultivar, whereas both genes were upregulated exclusively in the Sissy cultivar at 48 HAI. *HSF-B1* TFs have been demonstrated to play a pivotal role in the activation of defense priming in *Arabidopsis* (Pick et al., 2012). *TGA3* is a member of the TGA TF family known as regulators of *PR* genes since they constitutively interact with the non-expresser *PR* gene 1 (*NRP1*) (Jakoby et al., 2002), while the *tga3 Arabidopsis* mutant is defective in basal pathogen resistance (Kesarwani et al., 2007). This suggests that *TGA3*-*like* TFs play a central role in the activation of *PR* genes in the cv. Sissy. In contrast, plant-derived cytokinins promote *Arabidopsis* resistance to *Pst* DC3000 through *TGA3-*dependent activation of ARR2, a cytokinin-activated transcription factor (Choi et al., 2010). This supports further our results, as the main hub gene in the regulatory network of the Sissy 48 HAI-related module was a cytokinin dehydrogenase 7-encoding gene. Such cytokinin-related genes have been reported to support plant responses and resistance to necrotrophic pathogens (Li et al., 2022; Zhu et al., 2022).

Gene co-expression network analyses also have limitations, even if they provide valuable information about potential genes and their correlation, as they do not indicate the nature of the relationship between genes that are co-expressed. Thus, further analyses are necessary to define which genes are regulated by the identified TFs inducing Sissy tolerance to *M. laxa*, such as ChIP-seq (Chen et al., 2018), which can be integrated into our data to allow the identification of the target genes of TFs.

## Conclusions

Comparative transcriptomics is a powerful tool for identifying the key genes and pathways that are differentially expressed during pathogen invasion. In this study, the application of this technique provided a comprehensive view of the molecular mechanisms underlying the differential tolerance of the petals of the two pear cultivars, Sissy and Kristalli. Transcriptome profiling revealed cultivar-specific and time-dependent responses after *M. laxa* inoculation. In particular, GO terms and KEGG pathway enrichment analyses showed an earlier transcriptome reprogramming in Kristalli compared with the Sissy cultivar, whereas defense-related DEGs were mainly suppressed. DEGs involved in signal transduction, biosynthesis of secondary and primary metabolism, and other defense-related responses were mainly induced in the relatively tolerant Sissy cultivar at 48 HAI. These results allow us to better decipher the pear–*M. laxa* pathosystem identifying the main pathways involved in pear petal defense responses against *M. laxa*. The integration of a weighted gene co-expression network analysis with transcriptome profiling allowed the identification of six hub genes highly correlated to tolerance to *M. laxa*, including three *WRKY*s, one *TGA*, and one *HSF* TF, along with a cytokinin dehydrogenase-encoding gene, whose orthologs were already reported to be involved in crop pathogen resistance. The insights gained from our research may offer novel disease control strategies, such as new target genes for genome editing to develop new resistant and transgene-free pear cultivars.

## Data availability statement

The datasets presented in this study can be found in online repositories. The names of the repository/repositories and accession number(s) can be found below: https://www.ncbi.nlm.nih.gov/genbank/, PRJNA1049420.

## Author contributions

MA: Conceptualization, Data curation, Formal analysis, Investigation, Methodology, Software, Validation, Writing – original draft, Writing – review & editing. PT: Formal analysis, Investigation, Methodology, Writing – original draft, Writing – review & editing. AB: Formal analysis, Investigation, Writing – original draft. AD: Investigation, Writing – original draft. MM: Investigation, Writing – original draft. CD: Writing – review & editing. DT: Conceptualization, Methodology, Writing – original draft. EP: Conceptualization, Methodology, Writing – original draft. AM: Data curation, Formal analysis, Investigation, Validation, Writing – review & editing. LS: Writing – review & editing. AZ: Conceptualization, Data curation, Formal analysis, Methodology, Supervision, Validation, Writing – original draft, Writing – review & editing.
