# Supplementary material for: Comparative transcriptome profiling and co-expression network analysis uncover the key genes associated with pear petal defense responses against Monilinia laxa infection
Source: Front Plant Sci. 2024 Mar 7;15:1377937. doi: 10.3389/fpls.2024.1377937 (PMC10954844; doi:10.3389/fpls.2024.1377937)
Supplement: Supplementary file 1 [file DataSheet_1.pdf]

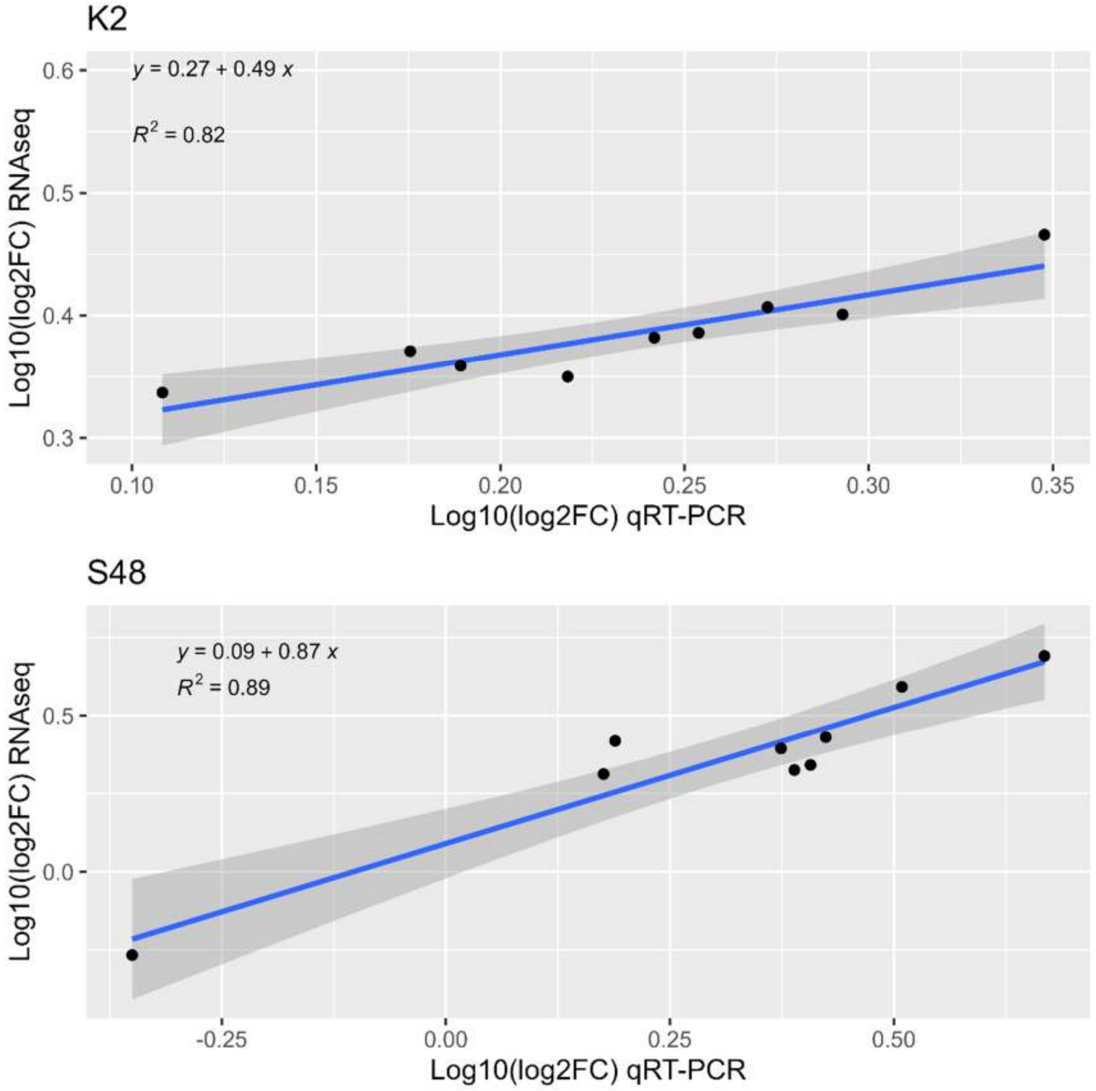

Supplementary Figure 1. Comparison of RNA-seq and qRT-PCR data at nine randomly selected genes for K2 and S48 comparison groups.
